# Response of Deep Subsurface Microbial Community to Different Carbon Sources and Electron Acceptors during ∼2 months Incubation in Microcosms

**DOI:** 10.3389/fmicb.2017.00232

**Published:** 2017-02-20

**Authors:** Lotta Purkamo, Malin Bomberg, Mari Nyyssönen, Lasse Ahonen, Ilmo Kukkonen, Merja Itävaara

**Affiliations:** ^1^VTT Technical Research Centre of FinlandEspoo, Finland; ^2^Geological Survey of FinlandEspoo, Finland; ^3^Department of Physics, University of HelsinkiHelsinki, Finland

**Keywords:** acetate, heterotrophy, autotrophy, deep biosphere, crystalline terrestrial bedrock, enrichment culture, competition, sulfate reducing bacteria

## Abstract

Acetate plays a key role as electron donor and acceptor and serves as carbon source in oligotrophic deep subsurface environments. It can be produced from inorganic carbon by acetogenic microbes or through breakdown of more complex organic matter. Acetate is an important molecule for sulfate reducers that are substantially present in several deep bedrock environments. Aceticlastic methanogens use acetate as an electron donor and/or a carbon source. The goal of this study was to shed light on carbon cycling and competition in microbial communities in fracture fluids of Finnish crystalline bedrock groundwater system. Fracture fluid was anaerobically collected from a fracture zone at 967 m depth of the Outokumpu Deep Drill Hole and amended with acetate, acetate + sulfate, sulfate only or left unamended as a control and incubated up to 68 days. The headspace atmosphere of microcosms consisted of 80% hydrogen and 20% CO_2_. We studied the changes in the microbial communities with community fingerprinting technique as well as high-throughput 16S rRNA gene amplicon sequencing. The amended microcosms hosted more diverse bacterial communities compared to the intrinsic fracture zone community and the control treatment without amendments. The majority of the bacterial populations enriched with acetate belonged to clostridial hydrogenotrophic thiosulfate reducers and *Alphaproteobacteria* affiliating with groups earlier found from subsurface and groundwater environments. We detected a slight increase in the number of sulfate reducers after the 68 days of incubation. The microbial community changed significantly during the experiment, but increase in specifically acetate-cycling microbial groups was not observed.

## Introduction

After the discovery of the existence of the deep, hot biosphere in the crustal setting over 20 years ago, we have been continuously amazed by the life thriving in these harsh and extreme environments (Gold, 1992; Whitman et al., 1998; McMahon and Parnell, 2014). These anoxic, highly reducing, saline environments are commonly nutrient-depleted and oligotrophic, while pressure and temperature increase with depth (Pedersen, 1993, 1997; Lovley and Chapelle, 1995; Amend and Teske, 2005; Fredrickson and Balkwill, 2006; Dong, 2008). Still, the carbon sources and energy substrates for the microbial life deep beneath the surface remain to be fully described. Traditional perception of the metabolism of deep-dwelling microbes is that they are chemolithotrophic using inorganic carbon and hydrogen as their carbon and energy source (Pedersen, 1993, 1997; Stevens and McKinley, 1995; Chapelle et al., 2002; Haveman and Pedersen, 2002; Amend and Teske, 2005; Lin et al., 2005a,b; Nealson et al., 2005). Autotrophs, such as methanogens and acetogens, acquire their carbon and energy sources from the deep Earth’s crust, while essential nutrients such as phosphorus and nitrogen can be derived from minerals or dissolved gas, respectively (Pedersen, 1997; Rogers et al., 1998; Bennett et al., 2001; Lin et al., 2005a). However, recent studies show that deep crystalline bedrock in Finland hosts diverse heterotrophic microbial communities in its ancient fracture fluids (Purkamo et al., 2013; Kietäväinen et al., 2014; Purkamo et al., 2015, 2016).

Organic carbon can originate from abiotic or biotic sources in the bedrock. Serpentinization, i.e., hydration of olivine in ultramafic rocks, leads to hydrogen generation and abiotic synthesis of organic matter (BR46; BR64; BR38; BR73; BR75). Many microbial groups can use acetate and contribute significantly to the total organic carbon content and carbon cycling of the deep biosphere (BR59; BR39). Acetate is a key molecule in carbon cycling and can be used by microbes involved in sulfur cycling, thus linking these two important elemental cycles together (BR51126). Sulfate reducers that are incomplete oxidizers produce acetate by metabolizing for example fatty acids, while other group of sulfate reducers completely oxidizes acetate to carbon dioxide (BR70). Aceticlastic methanogenic archaea can use acetate as an electron donor and/or a carbon source (e.g., BR12; BR13). Microbes are responsible for biological acetate production, either by autotrophy or by fermenting organic matter. Autotrophic acetogens in the deep subsurface use CO_2_ and H_2_ emanating from the mantle to produce acetate (BR59). Organic compounds, on the other hand, can either be transported from the surface deeper to the groundwater system by fluid circulation or after being buried and metamorphosed, remain trapped in the rock record (BR36; BR18; BR79). Organotrophic and fermenting microbes can decompose this more complex and sometimes recalcitrant organic matter to smaller compounds such as acetate (BR36; BR62,BR61; BR18; BR45). Recently, bacteriophages have been determined to be significantly impacting the deep crystalline bedrock biosphere by creating a viral shunt and release of organic material via bacterial cell lysis (BR37; BR60). Thus, using acetate as a representative of small organic carbon can help us to understand the organotrophic potential of microbial communities in the deep terrestrial biosphere.

The goal of this research was to study the effect of supplying an ample source of organic carbon to a microbial community inhabiting naturally oligotrophic groundwaters in deep Fennoscandian crystalline bedrock. In addition, we aimed to understand the role of sulfate reducers in these habitats and their ability to compete with methanogens and acetogens for substrates and electron donors. Since most microorganisms in the deep subsurface cannot be cultured, we applied molecular biology methods in order to reveal changes in community structure in groundwater samples enriched with an organic carbon source/electron donor (acetate), inorganic carbon source/electron acceptor (CO_2_) and/or electron acceptor (sulfate), and electron donor (H_2_) over an incubation period of over 2 months. DGGE fingerprinting was used first for rough characterization of the community structure and detecting the changes in the community during the incubation. As a nested PCR approach was required to produce sufficient amount of amplicons for DGGE, we decided to confirm the results and obtain a more refined characterization of the bacterial communities with high throughput amplicon sequencing. Acetate was chosen for organic carbon source for microbial communities for two reasons: (1) in anaerobic environments, such as the deep subsurface, acetate is a key intermediate in degradation of organic matter by for example sulfate reducers and (2), acetate could sustain heterotrophic communities which have previously been detected in the Fennoscandian shield bedrock environments (Itävaara et al., 2011; Purkamo et al., 2013, 2016; Nyyssönen et al., 2014). In addition, sulfate reducers can use acetate as a carbon source and as an electron donor. Sulfate reducing bacteria have been detected both in this fracture as well as in the drill hole fluids at the depth of the fracture zone, although the sulfate concentrations measured from this depth are less than 1 mg L^-1^ (Itävaara et al., 2011; Kietäväinen et al., 2013; Purkamo et al., 2016). Thus, the usability of sulfate as terminal electron acceptor for the microbial community in Outokumpu was also assessed. Autotrophic microbial groups in the microbial community were targeted with CO_2_ and H_2_ amendment in order to evaluate their potential in competing with heterotrophic microbes.

## Materials and Methods

### Sampling and Enrichment

The Outokumpu Deep Scientific Drill Hole pierces through Palaeoproterozoic rock formation comprising of metamorphic schists and skarns, serpentinized ophiolite-derived rocks and pegmatitic granite, reaching a depth of 2516 m. The fluids emanating from the 967 m fracture zone are saline: total dissolved solids are 13 g L^-1^, with Ca, Na and Cl as main dissolved components (Ahonen et al., 2011; Kietäväinen et al., 2013). Gas is abundantly present in the fluids, comprising mainly of methane at 967 m fracture depth (Kietäväinen et al., 2013). This drill hole provides access to intrinsic, millions of years old saline fracture fluids of Fennoscandian Shield when using appropriate sampling methods (Purkamo et al., 2013). The intrinsic microbial communities have been characterized from this fracture, and bacterial communities are comprised of phylotypes belonging to *Clostridia* and *Betaproteobacteria*. Archaeal populations at this fracture zone are dominated by *Hadesarchaea* and *Methanobacteriaceae* (Purkamo et al., 2016).

Fluid samples were collected in September 2009 from a fracture zone at the Outokumpu Deep Drill Hole. Sampling was optimized to obtain the indigenous microbial population from 967 m fracture zone with packer installation and sampling conducted as described on Purkamo et al. (2013). Briefly, two packers were positioned at 962 and 972 m depth flanking the fracture zone. The packers were expanded with tap water in order to seal the fracture zone and prevent water flow from other parts of the drill hole. After c. 4.6 m^3^ of fluid was pumped from the packer-isolated fracture zone, samples from indigenous fracture fluid were retrieved through a factory-clean polyamide tube. The fluid samples were collected in acid-washed, 120-mL serum bottles in a portable anaerobic chamber (MBRAUN, Garching, Germany), prepared under anoxic conditions as described in Purkamo et al. (2013). The sample bottles were first flushed with approximately 30 mL of fracture fluid in order to remove possible oxygen contamination of the bottle surfaces. After flushing, bottles were filled with 100 mL fracture fluid. The samples were amended with sodium acetate (final concentration 4 mM), sodium acetate and sodium sulfate (4 and 15 mM, respectively), only sodium sulfate (15 mM) or left unamended as controls. Vials were closed with butyl rubber stoppers and aluminum crimp caps (Sigma). The gas phase (20 mL) in the sealed bottles consisted of 80% hydrogen and 20% carbon dioxide, which was generated by gassing the headspace for approximately 1 min, and then adding a 20 mL overpressure of H_2_-CO_2_ gas mixture using a 50 mL syringe equipped with a needle pushed through the rubber stopper. All treatments were performed in triplicate and three similar sets were prepared, each to be sacrificed in one time point. Enrichments were incubated at 18°C, corresponding to the *in situ* temperature at 967 m in the drill hole, with slow rotation for 4, 32, or 68 days. In addition, biomass from a 100-mL sample of fracture water was collected on a Sterivex filter unit and immediately placed in dry ice in the field. A total of three of these zero-time point controls were collected and kept frozen at -80°C in the laboratory until DNA extraction.

### Biomass Collection and DNA Extraction

Following the incubation, biomass of the enrichments was collected from triplicates of each treatment with Sterivex filtration units (Millipore, Billercia, MA, USA) and immediately frozen to -80°C. DNA from the biomass of the enrichments was extracted with Mobio’s PowerWater DNA extraction kit (Mobio Laboratories, Inc. Carlsbad, CA, USA). Sterivex columns were thawed on ice and opened in a laminar flow hood using sterile pliers. Each filter was cut with a sterile scalpel to approximately 2 × 25 mm slices and transferred to a bead tube. DNA from the biomass was extracted according to the manufacturer’s protocol. DNA was eluted in 50 μl of molecular grade H_2_O. An extraction control without any sample was included in each extraction batch. Extracted DNA concentrations were measured with a NanoDrop-1000 spectrophotometer (Thermo Fisher Scientific, Waltham, MA, USA).

### Quantity of Total Bacteria, Sulfate Reducing Bacteria and Methanogens in the Microcosms

The numbers of 16S rRNA, *dsr*B and *mcr*A gene copies in the enrichments was determined with quantitative PCR (qPCR). The qPCR reactions (10 μl each) consisted of Kapa SYBR FAST 2x qPCR Master Mix for Roche LightCycler 480 (Kapa Biosystems, Woburn, MA, USA), 1.5, 2.5 or 2.5 pmol (16S rRNA gene, *dsr*B gene and mcrA gene, respectively) of each primer and 1 μl template DNA. The 16S ribosomal RNA genes were amplified with p1 and p2 primers (Muyzer et al., 1993), *dsr*B gene with primers DSRp2060f (5′-CAACATCGTYCAYACCCAGGG-3′) (Wagner et al., 1998) and DSR4r (5′-GTGTAGCAGTTACCGCA-3′) (Geets et al., 2006) and *mcr*A gene with primers ME1 (5′-GCMATGCARATHGGWATGTC-3′) (Hales et al., 1996) and ME3R (5′-TGTGTGAAWCCKACDCCACC-3′) (Nyyssönen et al., 2012). All qPCR reactions were conducted in triplicate and a negative template control was included in each run. The qPCR assays were performed with a LightCycler 480 (Roche Applied Science, Germany) using the following thermal protocol for 16S rRNA gene fragments: an initial denaturation step at 95°C for 10 min, 40 or 50 cycles of amplification with three steps: 15 s at 95°C, 30 s at 57°C and 30 s at 72°C. For *dsr*B genes, amplification was performed in 50 cycles of 10 s at 95°C, 35 s at 55°C and 30 s at 72°C. The *mcr*A genes were amplified with following protocol: 45 cycles of 10 s denaturation step at 95°C, 35 s annealing at 55°C, 30 s elongation step at 72°C. In addition, all qPCR programs contained a final elongation step for 3 min at 72°C. After the amplification, a melting curve analysis consisting of a denaturation step for 10 s at 95°C followed by an annealing step at 65°C for 1 min prior to a gradual temperature rise to 95°C at a rate of 0.11°C s^-1^ during which the fluorescence was continuously measured, was performed. Results of the melting curves were checked immediately after each run for primer dimer formation. Primer-dimer peaks were usually detected, but those were always at distinctly lower temperature (<72°C) as the main amplification products.

The copy number of each gene was estimated by comparing the amplification result to a standard dilution series reaching from 1.8 × 10^2^ to 1.8 × 10^8^ of plasmids containing the 16S rRNA gene of *Escherichia coli* ATCC 31608 (total bacterial number estimate) or 2.4 × 10^1^ to 2.4 × 10^7^ copies of plasmids containing the *dsr*B gene from *Desulfobulbus propionicus* DSM 2554 (estimated quantity of sulfate reducers) or plasmids containing the *mcr*A (dilution series from 5 to 5.03 × 10^6^ plasmids) of *Methanothermobacter thermoautotrophicus* strain DSM 1053.

### PCR-DGGE

The 16S rRNA gene -targeted PCR for bacteria employed a nested PCR approach, where the first round PCR primers fD1 (5′-AGAGTTTGATCCTGGCTCAG-3′) and rD1(5′-AAGGAGGTGATCCAGCC-3′) produce an almost full length 16S rRNA gene fragment (Weisburg et al., 1991), and the nested PCR primers p1(5′-CCTACGGGAGGCAGCAG-3′) and p2 (5′-ATTACCGCGGCTGCTGG-3′) a 193-bp fragment cover the V3 region of the gene (Muyzer et al., 1993). Primer p1 included a GC-clamp for the subsequent DGGE analysis. PCR amplification was performed in 50 μl reactions containing Dynazyme II buffer (10 mM Tris-HCl, pH 8.8, 1.5 mM MgCl_2_, 50 mM KCl, and 1% Triton-X 100), 1% formamide, 0.2 mM final concentration of each deoxynucleoside triphosphate dNTP, 20 pmol of each primer, 2 units of Dynazyme II polymerase enzyme (ThermoFisher Scientific, Waltham, MA, USA), and 1 or 2.5 μl of template (first PCR and nested PCR, respectively). The amplification program employed consisted of an initial denaturation step at 94°C for 5 min, 35 cycles of 1 min at 94°C, 1 min at 55°C, and 1 min at 72°C. A final elongation step of 10 min was performed at 72°C. Products from 16S rRNA gene-PCR were run in denaturing gradient gel electrophoresis (DGGE) as described in Purkamo et al. (2013). Distinctive DNA bands were cut out, the DNA extracted in 20 μl of molecular biology grade water (Sigma) overnight and frozen at -20°C before reamplification for sequencing using primers p1 and p2 as described above. PCR products were checked in 1% agarose gel and sent for sequencing to Macrogen Inc. (South Korea).

### High-Throughput Sequencing

The 16S rRNA gene amplicons were prepared from triplicate microcosms for each treatment and time point for high-throughput sequencing with the Ion Torrent PGM platform. Barcode attachment was done using PCR with 1,5 x MyTaq Red Mix according to the manufacturers instructions with primers 341f and 785r (20 mM each) (Herlemann et al., 2011) and 2 μl of template in 25 μl total reaction volume. Duplicate PCR reactions were prepared from each triplicate DNA extract in addition to the day 0 reference samples (i.e., the fracture water sample taken in the field), and with positive control (*Paracoccus denitrificans* DSM 413) and negative controls (PCR-grade water and extraction control sample). Duplicate PCR-products were combined and sequenced at Bioser Oulu (University of Oulu, Finland) using the 316 Chip Kit v2 with Ion PGM Template IA 500 and Ion PGM Hi-Q Sequencing kits (Thermo Fisher Scientific, Waltham, MA, USA).

### Accession Numbers

Retrieved sequences were deposited with the European nucleotide archive (ENA) database, DGGE band sequences with accession numbers LT634494-LT634570, and the Ion Torrent – produced amplicon sequences are deposited as data project PRJEB16746.

### Sequence Data Processing

Sequences obtained from DGGE analysis were aligned with relevant reference sequences retrieved from NCBI’s nucleotide database using the Geneious Pro software (v. 6.1.7, Biomatters Ltd, Auckland, New Zealand) with ClustalW and Muscle with default settings. Alignment was manually checked and edited, and phylogenetic trees were calculated using PhyML in Geneious Pro, applying the Jukes-Cantor 69 substitution model (Jukes and Cantor, 1969; Guindon and Gascuel, 2003).

The data retrieved from high-throughput sequencing (in fastq – format) was demultiplexed and quality screened with the MOTHUR software (v.1.36.0) (Schloss et al., 2009). Only the sequences with minimum length of 200 bp and average quality score 25 were retained. Sequences were aligned to the Silva reference alignment (release 123) (Quast et al., 2013). Preclustering was performed in order to remove sequences with possible sequencing errors. Chimeric sequences were identified with chimera.slayer command using Silva gold alignment as a reference and removed from the dataset. The unique sequences were classified using SILVA reference taxonomy (release 123)^1^ (Quast et al., 2013) and assigned to OTUs with phylotype command in MOTHUR. We used the alpha_diversity.py command for calculating the alpha diversity indices chao1, shannon and observed_otus using the QIIME software (MacQIIME v. 1.9.1) (Caporaso et al., 2010) using a biom-file constructed in MOTHUR. In addition, we calculated abundance coverage-based estimator (ACE), Good’s coverage and Shannon evenness metrics with MOTHUR using the command collect.single for the shared-file. Total richness and shared OTUs between sample replicates as well as samples were revealed by constructing venn-diagrams from the shared-file in MOTHUR.

### Statistical Analyses

Gel images of DGGE analysis were normalized, and the similarity of the sample banding profiles was calculated with Dice’s coefficient and UPGMA-dendrograms were constructed with the Bionumerics software package (v.5.10, Applied Maths, Sint-Martens-Latem, Belgium). Clustering of each treatment based on the relative amount of sequences in each OTU at each time point was analyzed with constrained Dice’s similarity index and UPGMA-dendrograms were constructed with the PAST program (v. 1.0, Hammer et al., 2001).

Richness and abundance data visualization and principal coordinates analysis (PCoA) of data was done with the R program using Vegan, Phyloseq and BiodiversityR packages (Kindt and Coe, 2005; McMurdie and Holmes, 2013; Oksanen et al., 2016).

## Results

### The Amount of Microbes in the Microcosm Enrichments

**FIGURE 1:**
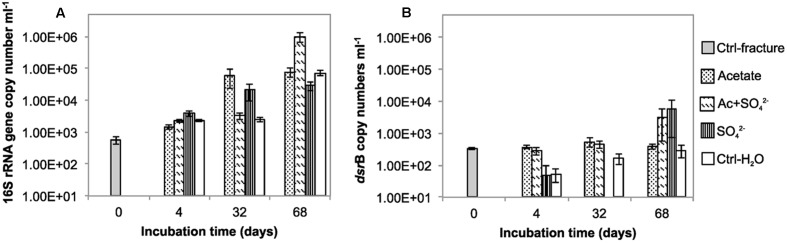
**The copy number of** **(A)** 16S rRNA gene **(B)** *dsrB* gene per mL in the microcosms incubated over a time span from 0 to 68 days.

The total amount of bacteria in the enrichments was evaluated with 16S rRNA gene-targeted qPCR. The mean 16S rRNA gene copy number in the untreated fracture zone fluid was 5.59 × 10^2^ copies mL^-1^ (S.E. 68.5) (**Figure 1A**; **Table 1**) and increased in all treatments and the unamended controls during the incubation. In acetate-amended microcosms, the 16S rRNA gene copy number increased during the incubation from 4 to 32 days by almost two orders of magnitude. In microcosms amended with acetate and sulfate, copy numbers increased after 32 days of incubation. After 68 days of incubation, the acetate and sulfate -treated fracture water sample had 1.01 × 10^6^ 16S rRNA copies (S.E. 3.63 × 10^5^) mL^-1^, which was the highest concentration of 16S rRNA gene copies observed throughout the enrichments. The microcosms amended only with sulfate had the highest amount of the 16S rRNA gene copies in the 4 days time point but only slight increase in copy numbers was detected in the later time points. In microcosms without any amendments the 16S rRNA gene copy numbers were also highest in the last time point at 68 days, with more than one order of magnitude increase from the second time point (from 2.52 × 10^3^ to 7.24 × 10^6^ of cells mL^-1^). Hence, using the 16S rRNA gene copy number counts as a proxy for total amount of bacteria, it is presumed that cell numbers increased in all treatments during the incubation time, including the control without any amendments. The qPCR efficiency of the 16S rRNA gene amplification E was 2.035, which corresponds to efficiency slightly over 100%, most likely due to some inhibition (Callbeck et al., 2013).

**Table 1:** The experimental setup, qPCR results and microbial community characteristics.

Experiment name^1^	Amendments		Incubation time	16S rRNA gene copy nr mL^-1^	*dsrB* gene copy nr mL^-1^	DGGE band affiliations	Most abundant OTUs from amplicon sequencing
	acetate	${SO}_{4}^{2-}$					
Acetate	+	-	4	1,48E+03	3,69E+02	*Dehalobacter. Desulfurella. unresolved*	*Dethiobacter* (31%)
Acetate	+	-	32	6,13E+04	5,36E+02	*Pseudomonadaceae. Mollicutes. Caulobacteraceae*	*Dethiobacter* (32%)
Acetate	+	-	68	7,83E+04	3,88E+02	*Caulobacter. Pseudomonadaceae*	*Brevundimonas* (72%)
Ac + ${SO}_{4}^{2-}$	+	+	4	2,31E+03	2,82E+02	*Dethiosulfatibacter. Sulfobacillus. Desulfurella. Mollicutes*	*Dethiobacter* (29%)
Ac + ${SO}_{4}^{2-}$	+	+	32	3,29E+03	4,49E+02	*Caulobacter. Desulfurella. Novosphingobium*	*Dethiobacter* (28%)
Ac + ${SO}_{4}^{2-}$	+	+	68	1,01E+06	3,12E+03	*Rhizobium. Cellulomonas. Stenotrophomonas*	*Rhizobium* (39%)
${SO}_{4}^{2-}$	-	+	4	3,91E+03	4,79E+01	*Desulfurella. Mollicutes*	*Dethiobacter* (40%)
${SO}_{4}^{2-}$	-	+	32	2,13E+04	nd	*Desulfurella. Mollicutes*	*Dethiobacter* (31%)
${SO}_{4}^{2-}$	-	+	68	2,91E+04	5,96E+03	*Pseudomonadaceae. Agrobacterium. Sulfobacillus. Desulfurella*	*Novosphingobium* (14%) *Nocardioides* (14%)
Ctrl-H_2_O	-	-	4	2,38E+03	5,38E+01	*Desulfurella. Mollicutes. Desulfotomaculum*	*Dethiobacter* (33%)
Ctrl-H_2_O	-	-	32	2,52E+03	1,66E+02	*Mollicutes*	*Dethiobacter* (31%)
Ctrl-H_2_O	-	-	68	7,24E+04	2,95E+02	*Agrobacterium. Arthrobacterium. Cellulomonas*	*Cellulomonas* (41%)
Ctrl-fracture^1^	-	-	0	5,59E+02	3,24E+02	*Mollicutes. Desulfurella*, sequences affiliating with plastids	Unclassified *Chloroplast* (77%) *Dethiobacter* (10%)


*^1^All experiments were done in microcosms except Ctrl-fracture, which represents sample from the intrinsic fracture fluid. nd, not detected.*

The copy number of *dsr*B genes in the microcosms was used as a proxy for the amount of sulfate reducing bacteria. The *dsr*B gene copy number in original fracture zone water was 3.24 × 10^2^ (S.E. 2.46 × 10^1^) copies mL^-1^, i.e., 58% of the amount of bacterial 16S rRNA gene copies (**Figure 1B**). In acetate only -amended treatments, enrichment time did not have significant effect on *dsr*B copy numbers as copy numbers remained at the same level with that of the original fracture fluid throughout the incubation period. Microcosms amended with acetate and sulfate had significant increase in the number of *dsr*B gene copies after 32 days of incubation. Greatest fluctuation in the number of *dsr*B gene copies was detected in the sulfate-amended microcosms, where copy numbers ranging from near or below the detection limit to 5.96 × 10^3^ (S.E. 5.22 × 10^3^) copies mL^-1^ after 68 days of incubation. However, this should be considered cautiously, since *dsr*B copies were only detected in one of the three replicate samples of this treatment. A slight increase was detected during the incubation in the control without amendments also in the *dsr*B gene copy numbers, as was the case with the total bacterial 16S rRNA gene copy numbers. The *dsr*B amplification efficiency E was 1.802 corresponding 80% efficacy.

We also attempted to quantify methanogens in the microcosms using *mcr*A gene, the marker gene for methanogenesis as a proxy. However, *mcr*A copy numbers were all below the detection limit of the qPCR assay (<5 copies mL^-1^).

### Changes in the Bacterial Community Structure during the Experiment

**FIGURE 2:**
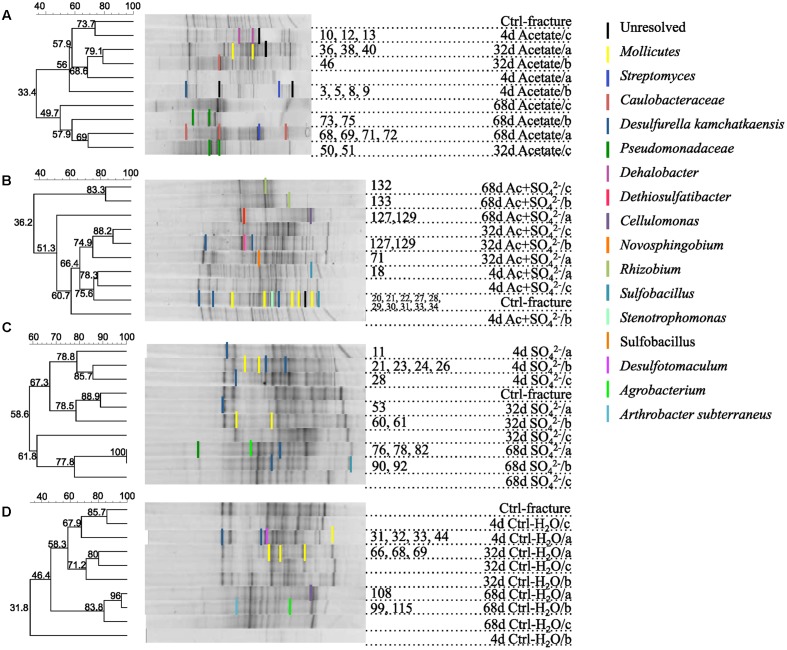
**DGGE fingerprints of the microbial communities in microcosms amended with** **(A)** acetate, **(B)** acetate + ${SO}_{4}^{2-}$, **(C)** ${SO}_{4}^{2-}$, or **(D)** left unamended. UPGMA dendrograms are based on similarity calculated with Dice’s coefficient. Numbers of the DGGE bands correspond the sequence numbers in the phylogenetic tree (see **Supplementary Figure S1**).

**FIGURE 3:**
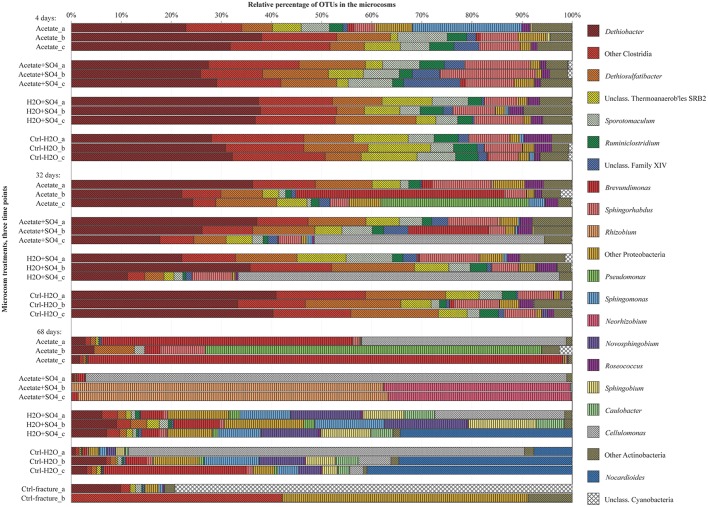
**Bacterial community structure in replicate microcosms with different treatments from three time points, determined with amplicon sequencing.** Only dominating OTUs are shown in genus level, others OTUs are combined into groups corresponding their phylum. *Firmicutes* are marked with diagonal lines, *Proteobacteria* with vertical lines and *Actinobacteria* with dots.

Bacterial communities in the different treatments over the span of the experiment were assessed with bacterial 16S rRNA gene DGGE and high-throughput amplicon sequencing. The microbial community structure in the microcosms changed during the incubation in all treatments (**Figures 2** and **3**, **Supplementary Figure S1**). Major changes were not observed in the community structure between the different treatments after 4 and 32 days of incubation (**Figures 2** and **3**). However, after 68 days the bacterial communities in the microcosms differed significantly from one another. There was a notable dispersion in the Shannon diversity index between the communities (**Table 2**).

**Table 2:** Ecological indices from amplicon sequencing.

Experiment	Incubation	No. of sequences^3^	OTUs	Shared OTUs		Coverage	Shannon	Chao1	ACE	OTUs/Chao1	OTUs/ACE	Shannon
					
	time (days)		observed	between replicates^4^	/total richness		H’					evenness
Acetate	4	7618	99	21	21%	99.8%	4.02	108.5	106.9	91%	93%	0.6
Acetate	32	12048	108	17	16%	99.8%	3.81	154.4	133.1	70%	81%	0.6
Acetate	68	5449	50	5	10%	99.7%	1.49	60.9	79.1	82%	63%	0.3
Ac + ${SO}_{4}^{2-}$	4	10334	89	23	26%	99.8%	3.77	116.1	109.7	77%	81%	0.6
Ac + ${SO}_{4}^{2-}$	32	3401	80	18	23%	99.3%	3.81	103.0	104.7	78%	76%	0.6
Ac + ${SO}_{4}^{2-}$	68	51286	43	2	5%	100.0%	1.71	48.0	51.2	90%	84%	0.3
${SO}_{4}^{2-}$	4	16782	121	25	21%	99.9%	3.65	160.4	141.3	75%	86%	0.5
${SO}_{4}^{2-}$	32	9763	100	18	18%	99.8%	3.76	113.9	112.6	88%	89%	0.6
${SO}_{4}^{2-}$	68	13287	98	38	39%	99.8%	4.16	113.0	118.9	87%	82%	0.6
Ctrl-H_2_O	4	22282	124	33	27%	99.9%	3.75	153.5	149.3	81%	83%	0.5
Ctrl-H_2_O	32	11428	97	19	20%	99.9%	3.36	108.3	110.9	90%	87%	0.5
Ctrl-H_2_O	68	20093	87	34	39%	99.9%	2.76	168.2	153.5	52%	57%	0.4
Ctrl-fracture	0	4659	48	5	10%	99.8%	1.62	57.0	54.1	84%	89%	0.3
Negative control^1^	-	80	8	na	na	98.8%	2.34	8.0	8.4	100%	95%	0.8
Positive control^2^	-	41983	5	na	na	100.0%	0.01	6.0	11.8	83%	42%	0.0


*^1^Combined data from PCR-blank and DNA extraction control. ^2^Amplified DNA from Paracoccus denitrificans. ^3^After quality control. ^4^Shared OTUs between sample replicates. na, not available.*

**FIGURE 4:**
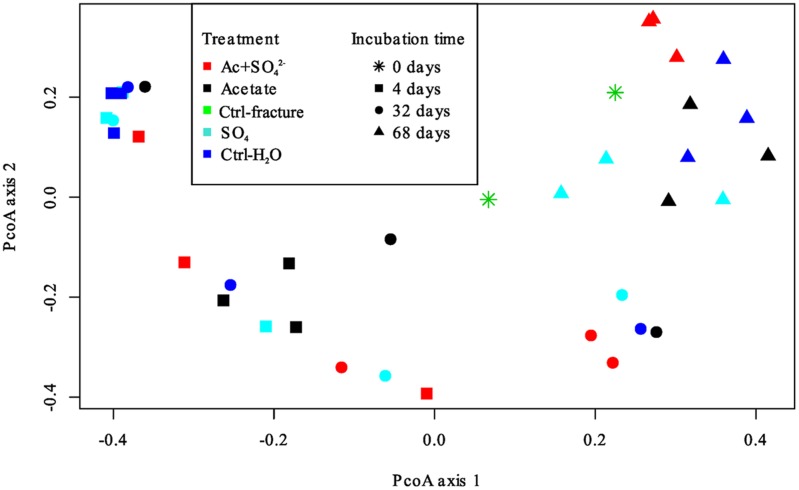
**Principal coordinates analysis plot of the microbial communities in the microcosms based on Bray-Curtis similarity model.** Axis 1 explained 26.8% and axis 2 15.4% of the variation in the data. Treatments are represented by different colors; incubation time is represented by different symbols.

The DGGE profiles of each treatment showed a decrease in the number of phylotypes (i.e., bands in the fingerprint of each community) during the incubation (**Figure 2**). According to the UPGMA trees based on the DGGE band positions of each community, the bacterial communities were broadly divided into two groups, one comprising of the intrinsic fracture fluid community and microcosms incubated for 4 and 32 days and the other the bacterial community after 68 days of incubation. Similarly, according to hierarchical clustering analysis, based on the relative amount of sequences in each OTU in each sample produced by amplicon sequencing, bacterial communities after 4 and 32 days of incubation resembled each other and the communities in the original fracture water and differed from the 68-day incubations (**Supplementary Figure S2**). Correspondingly, the microbial communities after 68 days of incubation clustered together in the PCoA based on amplicon sequencing data (**Figure 4**). The eigenvalues were 3.23 (axis 1) and 1.85 (axis 2) and these dimensions explained 26.8 and 15.4% of the variation in the data, respectively. Generally, samples tended to cluster based on the incubation time rather than the treatment in the PCoA.

The amount of observed OTUs generally diminished during the incubation (**Table 2**). After 4 days of incubation, the total number of OTUs in all treatments was 209. At this time point, there were no significant differences in the amount of shared OTUs between different treatments. Forty-four OTUs were shared between all microcosm communities. After 32 days of incubation, the number of OTUs decreased in all other treatments but those amended with acetate and sulfate. The number of shared OTUs between all treatments had decreased to 36 OTUs. After 68 days of incubation, the number of OTUs had decreased even more, to only 20 OTUs shared in all treatments. The shared OTUs between sequenced replicate microcosms was on average 21%, however, there were some samples that shared only a few OTUs between the replicates (acetate + sulfate treatment at 68 days) (**Table 2**). Good’s coverage estimate was close to 100% in all sequenced communities (**Table 2**). Based on the Chao1 and ACE estimates, on average 80% of the total richness and abundance in the microcosm communities was captured with the amplicon sequencing. The Shannon diversity index ranged from 1.49 to 4.16 between treatments and time points, with a predominant trend of lower diversity after longer incubation period. The exception was the microcosms amended only with sulfate, in which the diversity increased during the incubation period and the highest Shannon diversity H′ (4.16) was observed in the last time point in after 68 days. The evenness of the communities in the microcosms ranged from 0.3 to 0.6, whereas the original fracture fluid bacterial community was less even (0.3).

Sequences of selected DGGE bands were used as a rough characterization of the bacterial communities. The fingerprint of bacterial community in fracture fluid used as an inoculum for the microcosms comprised of several bands affiliating with *Mollicutes* in addition to two distinct bands closely related to *Deltaproteobacteria* (*Desulfurella*). Some sequences affiliated with plastids (**Table 1**, **Figure 2**), determined by their clustering in the maximum likelihood tree (**Supplementary Figure S1**). Based on the high-throughput amplicon sequencing data, the intrinsic bacterial community in the 967 m fracture zone fluid was dominated by sequences affiliating with chloroplasts in addition to clostridia and proteobacteria. A minor proportion of the community fell with *Dethiobacter* (**Figure 3**). However, if chloroplasts were filtered from the sequence data, the remaining bacterial community was very similar with those in the microcosms after 4 days of incubation. The bacterial community fingerprint of the fracture fluid was most similar with the bacterial communities after 4 and 32 days of incubation (**Figure 2**, **Supplementary Figure S2**).

In bacterial communities of the acetate-amended microcosms, DGGE band sequences affiliating with *Dehalobacter. Desulfurella* and *Mollicutes* were common after four and 32 days of incubation, whereas sequences related to *Caulobacteraceae* and *Pseudomonadaceae* dominated after 68 days (**Figure 2A**, **Supplementary Figure S1**). Based on amplicon sequencing, on average over 30% of the sequenced community at 4- and 32-day acetate-enrichments were affiliated with *Dethiobacter* (**Figure 4**). Otherwise the phylotypes in these communities resembled other *Clostridiales* and alphaproteobacterial 16S rRNA gene sequences.

The bacterial community of the microcosms enriched with acetate and sulfate, assessed from the sequenced DGGE bands, consisted of phylotypes affiliating with clostridial *Sulfobacillus* and *Dethiosulfatibacter*, proteobacterial *Desulfurella. Mollicutes*, and especially after 68 days, phylotypes affiliating with *Rhizobium* (**Figure 2B**, **Supplementary Figure S1**). Amplicon sequencing confirmed the dominance of clostridial phylotypes such as *Dethiobacter. Dethiosulfatibacter. Sporotomaculum, Thermoanaerobacterales* – affiliating SRB2 phylotype, *Ruminiclostridium* and clostridial Family XIV – related bacteria after 4 and 32 days of incubation in acetate and sulfate-amended microcosms, however, in one microcosm (replicate c) after 32 days of incubation *Cellulomonas* was dominant. After 68 days, the enrichment had a distinct community structure with phylotypes representing actinobacterial *Cellulomonas* and alphaproteobacterial *Rhizobiaceae*, Clostridia being virtually absent (**Figure 3**). In addition, the diversity of this community was significantly lower after 68 days (H′ 1.71) compared to that of earlier time points.

Microcosms amended with only sulfate hosted bacterial communities resembling those of the microcosms amended with acetate and sulfate. Phylotypes of sequenced DGGE bands from day 4 and day 32 affiliated with *Desulfurella* and *Mollicutes*, and phylotypes from 68 days affiliated with *Sulfobacillus. Desulfurella. Agrobacterium*, and *Pseudomonas* (**Figure 2C**, **Supplementary Figure S1**). Amplicon sequencing revealed that after 4 and 32 days, similar phylotypes belonging to *Clostridiales* that were also detected from the acetate + sulfate –amended microcosms, were the most dominant in solely sulfate-amended experiments, although in one replicate microcosm after 32 days of incubation, *Cellulomonas* phylotypes were dominating over *Clostridiales* (replicate c) (**Figure 3**). The bacterial community after 68 days in the sulfate-amended microcosms was the most diverse. On average, 60% of the phylotypes belonged to alphaproteobacterial groups such as *Sphingomonas. Sphingorhabdus. Sphingobium. Novosphingobium. Caulobacter*, and *Methylobacter*. Other phylotypes affiliated with Clostridia and Actinobacteria.

Microcosms left unamended hosted bacterial communities slightly dissimilar to the amended ones, especially at the last time point after 68 days. The communities detected with DGGE comprised of phylotypes affiliating with *Mollicutes. Actinobacteria* and deltaproteobacterial *Desulfurella* (**Figure 2D**). Clostridial phylotypes were most common after 4 and 32 days of incubation in the communities characterized with amplicon sequencing, but after 68 days, the community consisted of actinobacterial *Cellulomonas* and *Nocardioides* and of alphaproteobacterial phylotypes (**Figure 3**).

## Discussion

### The Changes in the Microbial Community Structure

Previous studies have reported taxonomically and functionally diverse microbial communities dwelling in methane-rich, oligotrophic and saline groundwaters in Outokumpu (Kietäväinen et al., 2013; Purkamo et al., 2013, 2016; Nyyssönen et al., 2014). Moreover, heterotrophic carbon assimilation by *Clostridia* was determined to be important for the microbial life throughout the deep drill hole water column (Purkamo et al., 2015). These data steered us to investigate further on the preferred carbon and energy sources of these microbial communities.

We provided different combinations of carbon sources, electron donors and electron acceptors to the intrinsic microbial community of the fracture fluid in order to identify the species benefiting from the amendments. Previous studies have shown that there are active sulfate reducers present in Outokumpu deep subsurface, although sulfate concentrations are low (<1 mg L^-1^ at 967 m depth) (Kietäväinen et al., 2013; Purkamo et al., 2016). The dominant sulfate reducers in Outokumpu affiliate with *Desulfotomaculum*, which are commonly found from both deep terrestrial and marine subsurface environments (e.g., Moser et al., 2005; Nakagawa et al., 2006; Aüllo et al., 2013; Bomberg et al., 2015; Purkamo et al., 2016). In this study we offered an easily utilizable organic carbon source together with suitable electron donor and acceptor for sulfate reducing bacteria. Our results showed an increase of *dsrB* gene copy numbers in acetate + sulfate-supplemented microcosms during the incubation period. This is in accordance with Pedersen (2012), who reported multiplication in total number of cells as well as in the most probable number of sulfate reducing bacteria in the first 30 days in in-situ flow cell cabinets amended with acetate. Although sulfate reducers were not detected with the bacterial community analyses, hydrogenotrophic thiosulfate reducers dominated the enrichments after 4 and 32 days. The detection of hydrogenotrophic bacteria and their dominance in the microcosms suggests that addition of hydrogen was in fact determining the microbial community structure more than the provided carbon source and electron acceptor. Particularly *Dethiobacter* was a major component of the bacterial communities in all microcosms, independent of whether the microcosms had received other amendments than H_2_ and CO_2_. *Dethiobacter* is described as a hydrogen-respiring organism using thiosulfate, elemental sulfur, and polysulfide as electron acceptors (Sorokin et al., 2008) and has previously been detected from several serpentinizing, ophiolithic rock and deep terrestrial subsurface environments (Brazelton et al., 2013; Suko et al., 2013; Tiago and Veríssimo, 2013; Crespo-Medina et al., 2014; Woycheese et al., 2015; Purkamo et al., 2016). Similar to our results, Crespo-Medina et al. (2014) reported that *Dethiobacter* was almost exclusively enriched in the microcosms originating from serpentinizing groundwater, independent of nutrient addition. To our knowledge, *Dethiobacter* have not been commonly found from marine deep subsurface. Additionally, unclassifiable members of Clostridial family XIV resembling *Anaerobranca* were detected in our enrichments. *Anaerobranca* –type microorganisms, like their distant relatives *Dethiobacter*, have the capacity to reduce thiosulfate to sulfide, in addition to the reduction of elemental sulfur. These alkaliphilic bacteria essentially produce acetate by fermenting more preferred, proteinaceous compounds (Wiegel, 2015). Therefore we assume that these organisms, including *Dethiobacter* that has previously been characterized also as one of the keystone genera of Outokumpu deep biosphere, are important in converting dead biomass to acetate and thus being major players in the carbon cycling in the deep crystalline bedrock habitat (Purkamo et al., 2016). Similarly, microbial communities have been shown to preferentially use proteinaceous substrates such as amino acids originating from dead microbial biomass, i.e., necromass, in deep marine subsurface (Lomstein et al., 2012; Lloyd et al., 2013).

Another hydrogen-respiring, thiosulfate-oxidizing species, *Dethiosulfatibacter* was present in the bacterial enrichments in this study. This microorganism has been detected at several other occasions from Outokumpu deep subsurface, but to our knowledge, not from other deep continental sites (Takii et al., 2007; Itävaara et al., 2011; Purkamo et al., 2013, 2016; Nyyssönen et al., 2014). Consequently, a *Dethiosulfatibacter* -affiliating OTU was described to be part of the core microbial community of Outokumpu deep subsurface with only a few other species (Purkamo et al., 2016). Other relatively abundant clostridial phylotypes in the first two time points, such as *Sporotomaculum* and *Ruminiclostridium* are also not sulfate reducers but gain energy by fermentation of organic matter (Yutin and Galperin, 2014; Rainey, 2015).

While the amount of total organic carbon in Outokumpu fracture fluids is around 6 mg mL^-1^, most of this is likely dissolved methane (80 vol-% in the fracture fluids) (Kietäväinen et al., 2013; Kietäväinen and Purkamo, 2015; Purkamo et al., 2016). Hence, methanogens could be responsible for production of this methane in deep subsurface of Outokumpu. In this study, aceticlastic methanogens were targeted with acetate amendment together with CO_2_ and H_2_. For comparison, other microcosms were amended with CO_2_ and H_2_ that would benefit specifically autotrophic methanogens and acetogens. However, methanogens were not detected in any of the microcosms. This is in agreement with Purkamo et al. (2016), where no methanogens were detected from this fracture and *Hadesarchaea* dominated the total archaeal community. Thus, we conclude that the heterotrophic community members, such as *Brevundimonas* -affiliating phylotypes whose relative abundance increased in the acetate-amended microcosms during the incubation, benefited from the acetate amendment. Alphaproteobacterial *Brevundimonas* has been identified previously in an enrichment experiment done with granitic bedrock fluids from Mizunami, Japan (Fukuda et al., 2010). A phylotype closely related to *Brevundimonas mediterranea* represented the majority of the sequences in enrichments amended with organic acids or with H_2_ and CH_4_. Equally to our study, Fukuda et al. (2010) detected other α-proteobacterial phylotypes in significant amounts in their organic acid -amended enrichments. One *Brevundimonas* strain was also isolated from deep, subpermafrost subsurface brine from the Lupin Mine, Canada, with both aerobic TSA-medium and anaerobic heterotrophic medium with H_2_ and CO_2_ amendment (Onstott et al., 2009). The high percentage of *Alphaproteobacteria* in the end of the experiment after 68 days of incubation in our study as well as in the other studies probably results from their expertise in long-term survival in oligotrophic environments (Abraham et al., 1999). The emergence of opportunistic microbial species, such as *Alphaproteobacteria* and *Actinobacteria* toward the end of the experiment might be due to their capacity to use dead biomass for carbon and energy.

The most significant change in the bacterial communities in this study was observed after 68 days. There was a substantial increase in *Cellulomonas* phylotypes in the microcosms after 32 and especially after 68 days of incubation. *Cellulomonas* and other actinobacteria have been recurrently isolated from other deep terrestrial subsurface settings (Chang et al., 2007; Finster et al., 2009; Wouters et al., 2013; Puente-Sánchez et al., 2014). Their enrichment might indicate a succession process where an increase in cell death of *Dethiobacter*-type of microorganisms would subsequently enhance the growth of typical detrivore microbes, such as fermenters like *Cellulomonas*. In the beginning of the incubation the excess H_2_ in the microcosms inhibited *Cellulomonas*, but after *Dethiobacter* and *Dethiosulfatibacter* –type of organisms consumed the H_2_, the partial pressure of the hydrogen dropped enough to enable the growth of these fermenters. The measured 16S rRNA gene copy numbers stayed on the original level or increased during the incubation period. Nevertheless, it is possible that the amount of detected 16S rRNA gene copies could originate to some extent from the dead microbial biomass (Fittipaldi et al., 2011). Therefore, we presume that the conditions in the microcosms were first more optimal for the clostridial hydrogenotrophic phylotypes but became gradually more suitable for *Cellulomonas* and *Alphaproteobacteria*.

Some phylotypes in enrichments represented unexpected bacterial groups: *Sphingorhabdus* –affiliating phylotypes composed 7–12% of the total bacterial community in the microcosms after 4 and 32 days of incubation. These bacteria contain carotenoid pigments that absorb light and are capable of anoxygenic photosynthesis (Kim et al., 2007; Jogler et al., 2013). OTUs affiliating with *Roseococcus* represented a minor component of the bacterial community. Roseococci also contain carotenoid pigments as well as bacteriochorophyll *a*, which is used as a light-harvesting antenna. Roseococci can also produce energy by oxidation of thiosulfate to sulfate, hence Roseococci can be defined as facultative photoheterotrophs (Yurkov, 2015). The microcosms were kept in the dark, so the existence of bacteria affiliating with phototrophic organisms in the microcosms mimicking the deep, dark terrestrial subsurface remains a mystery. We did not find any sequences affiliating with roseococci or *Sphingorhabdus* from the negative controls, thus we argue that these unexpected genera can originate either from previous contamination of the groundwater fluids (possibly during the drilling) or be a real phenomenon.

### Evaluation of the Used Community Detection Methods

The quantity of microbes was relatively low in the fracture fluids used as the inoculum as well as in the microcosms during the enrichment, which challenged the microbial community detection. The microbial community structure was first roughly characterized with DGGE community fingerprinting and traditional Sanger sequencing of the common phylotypes. Amplicon sequencing produced a reasonable amount of sequences, keeping in mind the low concentration of biomass of the samples. While amplicon sequencing gave a more detailed presentation of the bacterial community, the changes in the communities in the microcosms during the incubation were visible with the DGGE fingerprints. The use of nested PCR for the DGGE in order to produce enough material for the fingerprinting method is a possible source of bias (Yu et al., 2015). However, independent of the community characterization method, the microbial community structure was comparable at the order level. Especially phylotypes belonging to *Alphaproteobacteria* were detected in the microcosms with both of the community characterization methods. On the other hand, *Desulfurella* -affiliating phylotype was frequently detected with DGGE in the bacterial communities of the microcosms, but was absent in the amplicon sequencing results. In addition, sequences affiliating with Mollicutes were more frequently detected with DGGE than with amplicon sequencing. Similarly, Mollicutes were detected from the fracture fluids at the same depth characterized with DGGE using the same primers without nested PCR (Purkamo et al., 2013), as well as in amplicon sequencing of the intrinsic bacterial community of the fracture with amplicon sequencing, using fD1 and p2 –primers (Purkamo et al., 2016). Hence, we presume that the primers used in this study for amplicon sequencing may exclude Mollicutes to some level. A relatively large amount of sequences affiliating with chloroplasts in the fracture fluid could be a result of PCR bias. The low amount of template material is known to generate random fluctuations in priming efficiency and lead to variable microbial community fingerprints (Chandler et al., 1997). In addition, GC-rich template sequences have been shown to have higher affinity to amplification primers (Polz and Cavanaugh, 1998), but whether the amplicon sequencing primers used in this study have higher affinity to chloroplast 16S rRNA gene is not known.

## Conclusion

The deep subsurface is considered to be a fairly stable environment over long periods of time (Hoehler and Jørgensen, 2013), thus deep continental biosphere is providing a habitat for recalcitrant microbial life. In this study we used microcosms to study the prospective changes in the microbial community structure when abundant carbon sources, electron donors and electron acceptors were introduced. Based on the results obtained in this study, organic carbon is useful for the heterotrophic microbial groups in deep biosphere in Outokumpu. However, according to our results it appears that heterotrophic sulfate reducers or aceticlastic methanogens did not benefit from the acetate addition. Sulfate addition did not have a major effect on the number of sulfate reducers, although a minor increase in SRB marker gene copy numbers was detected. Our results show that microbial communities in the deep terrestrial crystalline bedrock subsurface are subject to transformation. The remaining bacteria in the communities toward the end of the experiment in this study are likely to ferment the organic matter for energy production as well as have the ability to use proteinaceous substrates. Overall, it is likely that the deep terrestrial subsurface microbial communities are continuously changing, depending on the available substrates and dominating metabolic processes at each time point. These changes can be cyclic, i.e., heterotrophic and fermenting microbes can produce substrates to other microbial groups that can take over when viruses or substrate limitation will decrease the numbers of heterotrophs. However, we can only detect a snapshot of these microbial communities and may miss some of the changes with only 68 days incubation period.

This work provides a basis for further studies for detecting the predominant carbon sources and cycling mechanisms in deep crystalline rock fractures. The data gathered here provides guidance for detailed studies exploring the microbial community changes through time, detecting the major carbon assimilation mechanisms and identifying the populations using specific energy and carbon metabolisms. As this study setup does not provide information about the functionality of the microbes, testing the physiological responses and activity of these microbial communities to different carbon sources will further elucidate carbon cycling in the deep Fennoscandian biosphere in the future.

## Author Contributions

LP, MB, and MN designed the experiments and collected the samples. All analyses were carried out by LP. LA assisted with sample collection and provided the geochemical metadata. IK provided funding and access to Outokumpu Deep Drill Hole. MI provided funding and assistance in experimental design. All authors contributed to the discussion of the results. Manuscript was written by LP with inputs from other authors.

## Conflict of Interest Statement

The authors declare that the research was conducted in the absence of any commercial or financial relationships that could be construed as a potential conflict of interest.

## Footnotes

1 https://www.arb-silva.de/documentation/release-123/

## Supplementary Material

**FIGURE 1 | Phylogenetic affiliations of the partial 16S rRNA gene sequences retrieved from DGGE community fingerprinting analysis in relation to cultured reference strains and 16S rRNA gene sequences of uncultured environmental bacteria.** Bootstrap support values were calculated from 1000 random repeats. Nodes with >40% bootstrap support are indicated. Scale bar shows the percent difference.

**FIGURE 2 | UPGMA dendrograms of the similarity of bacterial community structure in microcosms with different treatments, (A)** acetate amended, **(B)** acetate + ${SO}_{4}^{2-}$ 4 amended, **(C)** only with SO_4_-amended and **(D)** without any amendments, based on amplicon sequencing results. Clustering was calculated using Dice similarity index.
