# Supplementary figures and images for: Response of Deep Subsurface Microbial Community to Different Carbon Sources and Electron Acceptors during ∼2 months Incubation in Microcosms
Source: Front Microbiol. 2017 Feb 20;8:232. doi: 10.3389/fmicb.2017.00232 (PMC5316538; doi:10.3389/fmicb.2017.00232)

A

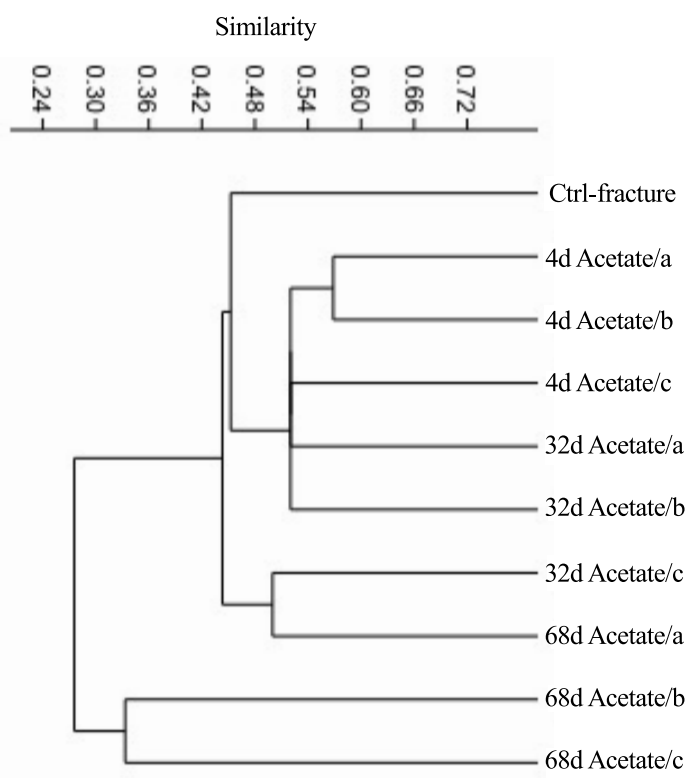

B

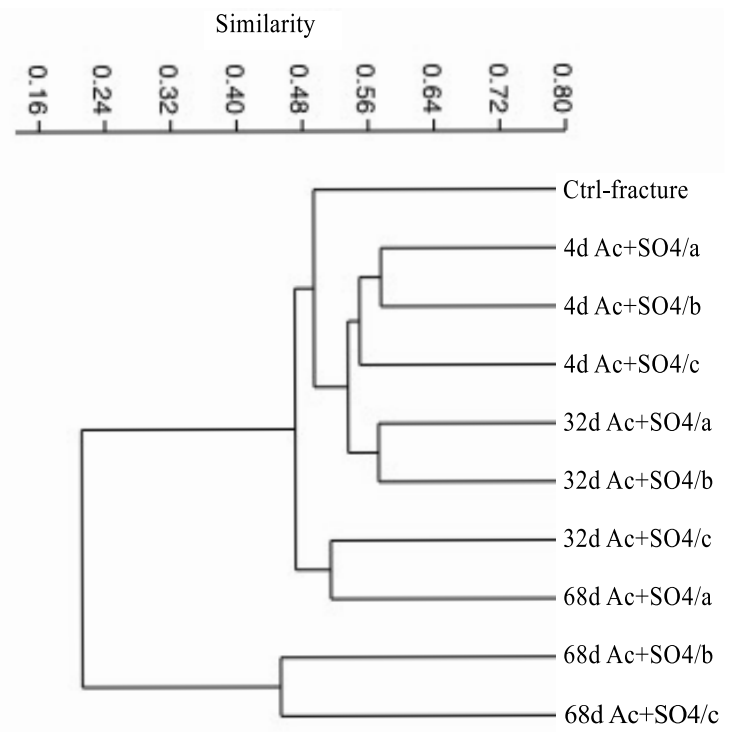

C

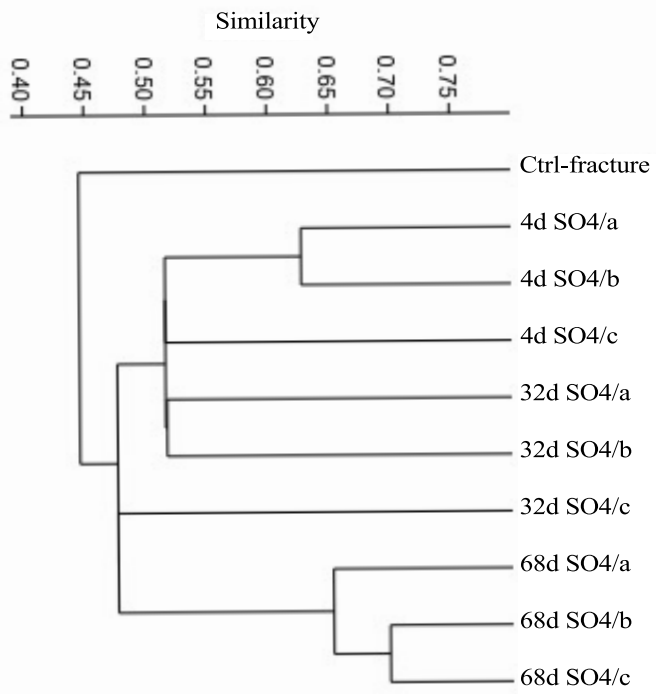

D

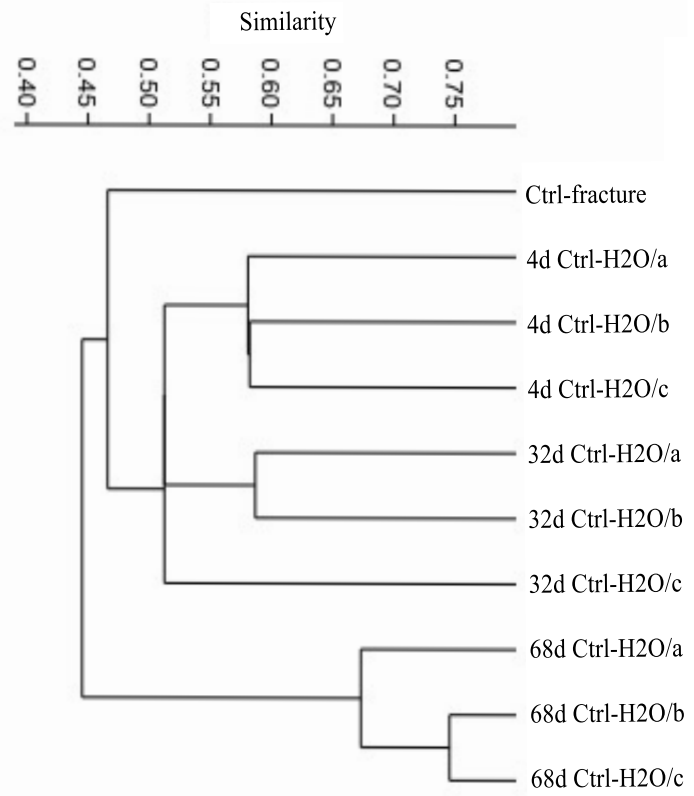

Supplement: Supplementary file 2 [file Image_2.PDF]
