# Supplementary material for: Response of Deep Subsurface Microbial Community to Different Carbon Sources and Electron Acceptors during ∼2 months Incubation in Microcosms
Source: Front Microbiol. 2017 Feb 20;8:232. doi: 10.3389/fmicb.2017.00232 (PMC5316538; doi:10.3389/fmicb.2017.00232)
Supplement: Supplementary file 1 [file Image_1.PDF]

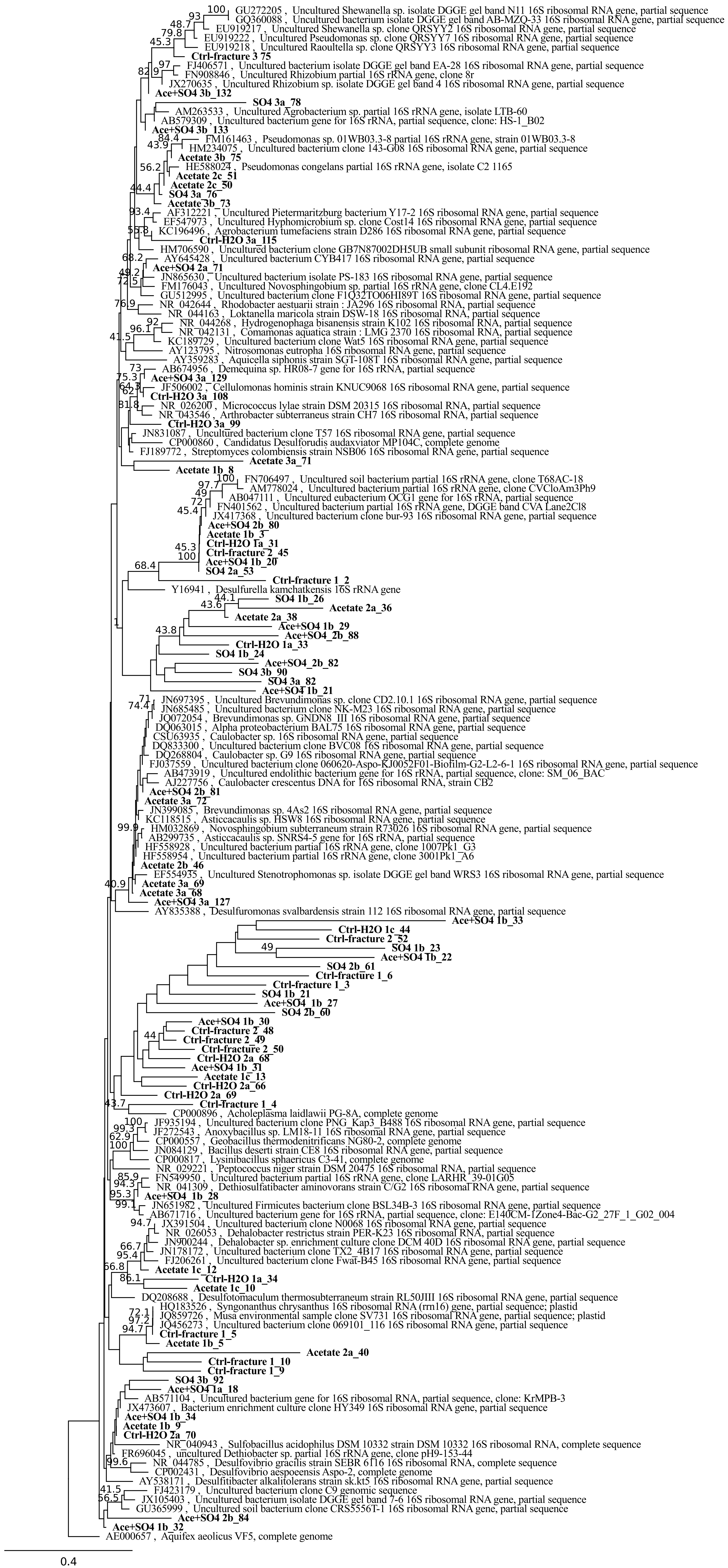
GU272205 , Uncultured Shewanella sp. isolate DGGE gel band N11 16S ribosomal RNA gene, partial sequence
GQ360088 , Uncultured bacterium isolate DGGE gel band AB-MZQ-33 16S ribosomal RNA gene, partial sequence
EU919217 , Uncultured Shewanella sp. clone QRSYY2 16S ribosomal RNA gene, partial sequence
EU919222 , Uncultured Pseudomonas sp. clone QRSYY7 16S ribosomal RNA gene, partial sequence
EU919218 , Uncultured Raoultella sp. clone QRSYY3 16S ribosomal RNA gene, partial sequence
Ctrl-fracture 3_75
FJ406571 , Uncultured bacterium isolate DGGE gel band EA-28 16S ribosomal RNA gene, partial sequence
FN908846 , Uncultured Rhizobium partial 16S rRNA gene, clone 8r
JX270635 , Uncultured Rhizobium sp. isolate DGGE gel band 4 16S ribosomal RNA gene, partial sequence
Ace+SO4 3b_132
SO4 3a_78
AM263533 , Uncultured Agrobacterium sp. partial 16S rRNA gene, isolate LTB-60
AB579309 , Uncultured bacterium gene for 16S rRNA, partial sequence, clone: HS-1_B02
Ace+SO4 3b_133
FM161463 , Pseudomonas sp. 01WB03.3-8 partial 16S rRNA gene, strain 01WB03.3-8
HM234075 , Uncultured bacterium clone 143-G08 16S ribosomal RNA gene, partial sequence
Acetate 3b_75
HE588024 , Pseudomonas congelans partial 16S rRNA gene, isolate C2 1165
Acetate 2c_51
Acetate 2c_50
SO4 3a_76
Acetate 3b_73
AF312221 , Uncultured Pietermaritzburg bacterium Y17-2 16S ribosomal RNA gene, partial sequence
EF547973 , Uncultured Hyphomicrobium sp. clone Cost14 16S ribosomal RNA gene, partial sequence
KC196496 , Agrobacterium tumefaciens strain D286 16S ribosomal RNA gene, partial sequence
Ctrl-H2O 3a_115
HM706590 , Uncultured bacterium clone GB7N87002DH5UB small subunit ribosomal RNA gene, partial sequence
AY645428 , Uncultured bacterium CYB417 16S ribosomal RNA gene, partial sequence
Ace+SO4 2a_71
JN865630 , Uncultured bacterium isolate PS-183 16S ribosomal RNA gene, partial sequence
FM176043 , Uncultured Novosphingobium sp. partial 16S rRNA gene, clone CL4.E192
GU512995 , Uncultured bacterium clone F1Q32TO06HI89T 16S ribosomal RNA gene, partial sequence
NR_042644 , Rhodobacter aestuarii strain : JA296 16S ribosomal RNA, partial sequence
NR_044163 , Loktanella maricola strain DSW-18 16S ribosomal RNA, partial sequence
NR_044268 , Hydrogenophaga bisanensis strain K102 16S ribosomal RNA, partial sequence
NR_042131 , Comamonas aquatica strain : LMG 2370 16S ribosomal RNA, partial sequence
KC189729 , Uncultured bacterium clone Wat5 16S ribosomal RNA gene, partial sequence
AY123795 , Nitrosomonas eutropha 16S ribosomal RNA gene, partial sequence
AY359283 , Aquicella siphonis strain SGT-108T 16S ribosomal RNA gene, partial sequence
AB674956 , Demequina sp. HR08-7 gene for 16S rRNA, partial sequence
Ace+SO4 3a_129
JF506002 , Cellulomonas hominis strain KNUC9068 16S ribosomal RNA gene, partial sequence
Ctrl-H2O 3a_108
NR_026200 , Micrococcus lylae strain DSM 20315 16S ribosomal RNA, partial sequence
NR_043546 , Arthrobacter subterraneus strain CH7 16S ribosomal RNA, partial sequence
Ctrl-H2O 3a_99
JN831087 , Uncultured bacterium clone T57 16S ribosomal RNA gene, partial sequence
CP000860 , Candidatus Desulforudis audaxviator MP104C, complete genome
FJ189772 , Streptomyces colombiensis strain NSB06 16S ribosomal RNA gene, partial sequence
Acetate 3a_71
Acetate 1b_8
FN706497 , Uncultured soil bacterium partial 16S rRNA gene, clone T68AC-18
AM778024 , Uncultured bacterium partial 16S rRNA gene, clone CVCloAm3Ph9
AB047111 , Uncultured eubacterium OCG1 gene for 16S rRNA, partial sequence
FN401562 , Uncultured bacterium partial 16S rRNA gene, DGGE band CVA Lane2Cl8
JX417368 , Uncultured bacterium clone bur-93 16S ribosomal RNA gene, partial sequence
Ace+SO4 2b_80
Acetate 1b_3
Ctrl-H2O 1a_31
Ctrl-fracture 2_45
Ace+SO4 1b_20
SO4 2a_53
Ctrl-fracture 1_2
Y16941 , Desulfurella kamchatkensis 16S rRNA gene
SO4 1b_26
Acetate 2a_36
Acetate 2a_38
Ace+SO4 1b_29
Ace+SO4_2b_88
Ctrl-H2O 1a_33
SO4 1b_24
Ace+SO4_2b_82
SO4 3b_90
SO4 3a_82
Ace+SO4 1b_21
JN697395 , Uncultured Brevundimonas sp. clone CD2.10.1 16S ribosomal RNA gene, partial sequence
JN685485 , Uncultured bacterium clone NK-M23 16S ribosomal RNA gene, partial sequence
JQ072054 , Brevundimonas sp. GNDN8_III 16S ribosomal RNA gene, partial sequence
DQ063015 , Alpha proteobacterium BAL75 16S ribosomal RNA gene, partial sequence
CSU63935 , Caulobacter sp. 16S ribosomal RNA gene, partial sequence
DQ833300 , Uncultured bacterium clone BVC08 16S ribosomal RNA gene, partial sequence
DQ268804 , Caulobacter sp. G9 16S ribosomal RNA gene, partial sequence
FJ037559 , Uncultured bacterium clone 060620-Aspo-KJ0052F01-Biofilm-G2-L2-6-1 16S ribosomal RNA gene, partial sequence
AB473919 , Uncultured endolithic bacterium gene for 16S rRNA, partial sequence, clone: SM_06_BAC
AJ227756 , Caulobacter crescentus DNA for 16S ribosomal RNA, strain CB2
Ace+SO4 2b_81
Acetate 3a_72
JN399085 , Brevundimonas sp. 4As2 16S ribosomal RNA gene, partial sequence
KC118515 , Asticcacaulis sp. HSW8 16S ribosomal RNA gene, partial sequence
HM032869 , Novosphingobium subterraneum strain R73026 16S ribosomal RNA gene, partial sequence
AB299735 , Asticcacaulis sp. SNRS4-5 gene for 16S rRNA, partial sequence
HF558928 , Uncultured bacterium partial 16S rRNA gene, clone 1007Pk1_G3
HF558954 , Uncultured bacterium partial 16S rRNA gene, clone 3001Pk1_A6
Acetate 2b_46
EF554935 , Uncultured Stenotrophomonas sp. isolate DGGE gel band WRS3 16S ribosomal RNA gene, partial sequence
Acetate 3a_69
Acetate 3a_68
Ace+SO4 3a_127
AY835388 , Desulfuromonas svalbardensis strain 112 16S ribosomal RNA gene, partial sequence
Ace+SO4 1b_33
Ctrl-H2O 1c_44
Ctrl-fracture 2_52
SO4 1b_23
Ace+SO4 1b_22
SO4 2b_61
Ctrl-fracture 1_6
Ctrl-fracture 1_3
SO4 1b_21
Ace+SO4_1b_27
SO4 2b_60
Ace+SO4 1b_30
Ctrl-fracture 2_48
Ctrl-fracture 2_49
Ctrl-fracture 2_50
Ctrl-H2O 2a_68
Ace+SO4 1b_31
Acetate 1c_13
Ctrl-H2O 2a_66
Ctrl-H2O 2a_69
Ctrl-fracture 1_4
CP000896 , Acholeplasma laidlawii PG-8A, complete genome
JF935194 , Uncultured bacterium clone PNG_Kap3_B488 16S ribosomal RNA gene, partial sequence
JF272543 , Anoxybacillus sp. LM18-11 16S ribosomal RNA gene, partial sequence
CP000557 , Geobacillus thermodenitrificans NG80-2, complete genome
JN084129 , Bacillus deserti strain CE8 16S ribosomal RNA gene, partial sequence
CP000817 , Lysinibacillus sphaericus C3-41, complete genome
NR_029221 , Peptococcus niger strain DSM 20475 16S ribosomal RNA, partial sequence
FN549950 , Uncultured bacterium partial 16S rRNA gene, clone LARHR_39-01G05
NR_041309 , Dethiosulfatibacter aminovorans strain C/G2 16S ribosomal RNA gene, partial sequence
Ace+SO4_1b_28
JN651982 , Uncultured Firmicutes bacterium clone BSL34B-3 16S ribosomal RNA gene, partial sequence
AB671716 , Uncultured bacterium gene for 16S rRNA, partial sequence, clone: E140CM-1Zone4-Bac-G2_27F_1_G02_004
JX391504 , Uncultured bacterium clone N0068 16S ribosomal RNA gene, partial sequence
NR_026053 , Dehalobacter restrictus strain PER-K23 16S ribosomal RNA, partial sequence
JN900244 , Dehalobacter sp. enrichment culture clone DCM 40D 16S ribosomal RNA gene, partial sequence
JN178172 , Uncultured bacterium clone TX2_4B17 16S ribosomal RNA gene, partial sequence
FJ206261 , Uncultured bacterium clone Fwat-B45 16S ribosomal RNA gene, partial sequence
Acetate 1c_12
Ctrl-H2O 1a_34
Acetate 1c_10
DQ208688 , Desulfotomaculum thermosubterraneum strain RL50JIII 16S ribosomal RNA gene, partial sequence
HQ183526 , Syngonanthus chrysanthus 16S ribosomal RNA (rrn16) gene, partial sequence; plastid
JQ859726 , Musa environmental sample clone SV731 16S ribosomal RNA gene, partial sequence; plastid
JQ456273 , Uncultured bacterium clone 069101_116 16S ribosomal RNA gene, partial sequence
Ctrl-fracture 1_5
Acetate 1b_5
Acetate 2a_40
Ctrl-fracture 1_10
Ctrl-fracture 1_9
SO4 3b_92
Ace+SO4 1a_18
AB571104 , Uncultured bacterium gene for 16S ribosomal RNA, partial sequence, clone: KrMPB-3
JX473607 , Bacterium enrichment culture clone HY349 16S ribosomal RNA gene, partial sequence
Ace+SO4 1b_34
Acetate 1b_9
Ctrl-H2O 2a_70
NR_040943 , Sulfobacillus acidophilus DSM 10332 strain DSM 10332 16S ribosomal RNA, complete sequence
FR696045 , uncultured Dethiobacter sp. partial 16S rRNA gene, clone pH9-153-44
NR_044785 , Desulfovibrio gracilis strain SEBR 6116 16S ribosomal RNA, complete sequence
CP002431 , Desulfovibrio aespoeensis Aspo-2, complete genome
AY538171 , Desulfitibacter alkalitolerans strain sk.kt5 16S ribosomal RNA gene, partial sequence
FJ423179 , Uncultured bacterium clone C9 genomic sequence
JX105403 , Uncultured bacterium isolate DGGE gel band 7-6 16S ribosomal RNA gene, partial sequence
GU365999 , Uncultured soil bacterium clone CRS5556T-1 16S ribosomal RNA gene, partial sequence
Ace+SO4 2b_84
Ace+SO4 1b_32
AE000657 , Aquifex aeolicus VF5, complete genome
0.4
